# COVID-19-related stigma and its sociodemographic correlates: a comparative study

**DOI:** 10.1186/s12992-021-00705-4

**Published:** 2021-05-07

**Authors:** Yuan Yuan, Yan-Jie Zhao, Qing-E Zhang, Ling Zhang, Teris Cheung, Todd Jackson, Guo-Qing Jiang, Yu-Tao Xiang

**Affiliations:** 1 Chongqing Mental Health Center, Chongqing, China; 2 Unit of Psychiatry, Department of Public Health and Medicinal Administration, & Institute of Translational Medicine, Faculty of Health Sciences, University of Macau, Macao SAR, China; 3 Centre for Cognitive and Brain Sciences, University of Macau, Macao SAR, China; 4 Institute of Advanced Studies in Humanities and Social Sciences, University of Macau, Macao SAR, China; 5 The National Clinical Research Center for Mental Disorders & Beijing Key Laboratory of Mental Disorders Beijing Anding Hospital & the Advanced Innovation Center for Human Brain Protection, School of Mental Health, Capital Medical University, Beijing, China; 6 School of Nursing, Hong Kong Polytechnic University, Hong Kong SAR, China; 7 Department of Psychology, University of Macau, Taipa, Macau SAR, China

**Keywords:** COVID-19 survivors, Stigma

## Abstract

**Background:**

Coronavirus disease 2019 (COVID-19) is closely associated with physical and mental health problems; however, little is known about the severity of stigma caused by COVID-19 among its survivors. Thus, the aim of this study was to compare differences in stigma experiences of COVID-19 survivors versus healthy controls after the COVID-19 outbreak peak in China.

**Methods:**

This cross-sectional study comprised 154 COVID-19 survivors and 194 healthy controls recruited through consecutive and convenience sampling methods, respectively. COVID-19 related stigma was measured by the Social Impact Scale (SIS). Stigma differences between the two groups were compared with analysis of covariance (ANCOVA) and a generalized linear model (GLM) was used to identify independent correlates of COVID-19-related stigma in this study.

**Results:**

Compared with healthy controls, COVID-19 survivors reported more overall stigma (*F*_(1,347)_ = 60.82, *p* < 0.001), and stigma in domains of social rejection (*F*_(1,347)_ = 56.54, *p* < 0.001), financial insecurity (*F*_(1,347)_ = 19.96, *p* < 0.001), internalized shame (*F*_(1,347)_ = 71.40, *p* < 0.001) and social isolation (*F*_(1,347)_ = 34.73, *p* < 0.001). Status as a COVID-19 survivor, having family members infected with COVID-19, being married, economic loss during the COVID-19 pandemic, and depressive symptoms were positively associated with higher overall stigma levels (all *p* values < 0.05).

**Conclusion:**

COVID-19-related stigma is commonly experienced among COVID-19 survivors even though the outbreak has been well-contained in China. Routine assessment of stigma experiences should be conducted on COVID-19 survivors and appropriate psychological assistance, public education, and anti-stigma campaigns and policies should be enforced to reduce stigma within this vulnerable subpopulation.

## Introduction

Coronavirus disease 2019 (COVID-19) was first reported in Wuhan, Hubei province, China at the end of 2019, and subsequently emerged in other parts of the world [66, 67]. COVID-19 was eventually declared to be a pandemic on 11 March 2020 by the World Health Organization (WHO) [69]. By early November 2020, around 50 million individuals had been infected [28], of whom, over 32 million have recovered from this disease [28]. In China the COVID-19 outbreak has been well controlled nationwide, except for occasional cases imported from overseas since early 2021 [13, 15].

Individuals suffering from COVID-19 may also have common psychiatric comorbidities and related problems such as depression, anxiety, stress and stigma [17, 26, 31, 48, 53, 54, 61]. Stigma refers to disapproval of or negative attitudes toward persons with certain characteristics or diseases that distinguish these persons from other members of society [23, 64]. In contrast, discrimination refers to the act of identifying and treating members of stigmatized groups unfairly compared to members of majority groups [4, 5, 9]. Persons who perceive they are being stigmatized may report guilt, self-blaming behaviour, self-depreciation, self-isolation, low self-esteem, and being excluded or ignored by others [35]. Stigma is closely associated with mental health problems particularly depression. Previous studies have found strong positive associations between stigma and depression; depression and stigma also have certain shared symptoms including feelings of guilt, self-blame, and low self-esteem [12, 52].

During the COVID-19 pandemic, stigma has become a public health challenge. COVID-19 related stigma refers to a disapproving or negative self-attitude, stemming from being infected with, or having close contacts with COVID-19, that results in “a spoiled identity” [41]. Recent findings suggest that some COVID-19 survivors and their family members are rejected by their neighbors, landlords and even employers in China [10]. Certain sub-populations such as persons suspected of having COVID-19, discharged individuals from quarantine, and people returning from overseas also experience various forms of stigmatization including social exclusion, stereotyping and being insulted [2]. Individuals suffering from social stigma may feel ashamed of themselves, and experience self-condemning behaviour or persistent fear of contacting their relatives and friends [10, 17]. For instance, Duan et al. [17] found that 16% of Hubei province residents reported being stigmatized during the peak of the COVID-19 outbreak and felt ashamed, blameworthy and shunned simply because Hubei was the COVID-19 epicenter. Another study from Vietnam found that 18% of healthcare workers felt unsafe to work in healthcare facilities after they undertook quarantine; 10% felt blameworthy by relatives and friends and 34% avoided contacts with neighbors or others in the community due to feeling stigmatized [16].

COVID-19 related stigma can lead to a range of negative consequences such as psychological stress [2], discrimination [18, 39], health-related violence [8, 42] and, worst of all, suicide [56] for affected populations including COVID-19 survivors, suspected or quarantined cases, and people returning from overseas. In addition, COVID-19-related stigma [25] could become a barrier to the control and prevention of COVID-19 because people who experience high levels of stigma are less likely to disclose their health status [2], thus deterring professional help-seeking behaviors [17], and refusing to take COVID-19 tests [19]. As stated by the WHO director-general Dr. Tedros Adhanom Ghebreyesus, “*stigma, to be honest, is more dangerous than the [corona] virus itself”* [68].

To date, some commentaries and reviews of COVID-19-related stigma have been published [29, 43, 50], but relevant quantitative studies are scant. Thus far, three studies focused on COVID-19-related stigma in healthcare workers [16, 51, 74], and one study investigating stigma in Hubei residents [17] have concluded these groups experience high stigma levels. A qualitative study has also assessed COVID-19-related stigma in two COVID-19 survivors and five family members [2].

Stigma related to infectious diseases is related to clinical features of such diseases and socio-cultural factors [33]. For instance, hepatitis A survivors rarely suffer from stigma yet hepatitis B and C survivors often experience high stigma levels due to more serious long-term effects [14]. Stigma related to infectious diseases is also common in East Asian countries and territories [33]. Previous studies found severe acute respiratory syndrome (SARS) survivors suffered from elevations in mental health problems including stigma in the post-SARS era [40, 57]. Thus, it is reasonable to assume that COVID-19 survivors experience more stigma than do uninfected peers.

Because COVID-19 is caused by a novel virus, it is not clear how widespread or severe stigma is among survivors. To date, no studies have compared COVID-19-related stigma between COVID-19 survivors and healthy controls. Therefore, the aim of this study was to compare COVID-19-related stigma between COVID-19 survivors and healthy controls living in the same region of China and to identify correlates of stigma experiences in these groups.

## Methods

### Study setting and participants

This was a cross-sectional, comparative study conducted between May 27 and September 4, 2020 at Chongqing Mental Health Center (CMHC), a setting that is responsible for follow-up mental health assessments of all COVID-19 survivors in Chongqing Municipality, China. Patients were eligible if they were 1) COVID-19 survivors in Chongqing, China; 2) aged 18 years or above; and 3) able to understand the purpose and contents of the assessment. All COVID-19 survivors attending the follow-up clinic at CMHC were consecutively invited to participate in this study. For the control group (healthy controls hereafter), eligibility criteria were the same except a COVID-19 diagnosis was absent. Healthy controls were recruited via convenience sampling of local community-dwelling residents in Chongqing. The study protocol was approved by the ethics committee of the Chongqing Mental Health Center. All participants provided online informed consent prior to participation.

### Assessment tools

The assessment was conducted by a research psychiatrist in a consultation room after participants attended their follow-up assessment. A data collection form was used to collect participant demographic data and clinical characteristics (gender, age, education level, marital status, living circumstances, occupation, perceived economic status, perceived health status). Participants were also asked whether they (1) had any family members infected with COVID-19; 2) perceived online mental health services to be helpful; 3) experienced economic loss during the COVID-19 outbreak; and 4) frequently received information about COVID-19 through social media.

Severity of fatigue was assessed using a fatigue numeric rating scale, which ranged from ‘0’ (no fatigue) to ‘10’ (extreme fatigue) [22]. The nine-item Patient Health Questionnaire (PHQ-9) - Chinese version assessed severity of depressive symptoms [58, 72]. Total PHQ-9 scores range from 0 to 27, with higher scores indicating more severe depressive symptoms.

Experiences of perceived stigma from COVID-19 were measured using the Social Impact Scale (SIS), a widely used 24-item measure of stigmatization used for patients with major medical conditions and infectious diseases such as the human immunodeficiency virus (HIV) [20]. The SIS is a generic stigma scale that can be used for different populations affected by the COVID-19 pandemic. Although healthy controls in the study were not infected with COVID-19, they were also greatly affected widespread quarantine measures during the pandemic such as suspended travel, limited outdoor activities, and school closures. The SIS covers 4 domains, including social rejection (9 items), financial insecurity (3 items), internalized shame (5 items), and social isolation (7 items). Each SIS item is rated from ‘1’ (strongly disagree) to ‘4’ (strongly agree), with total scores ranging from 24 to 96. Higher scores indicate more severe stigma. The Chinese version of the SIS has been validated with good psychometric properties [47].

### Statistical analysis

Data were analyzed using Statistical Analysis System (SAS) software, University Edition (SAS Institute Inc., Cary, North Carolina, U.S.). Comparisons of demographics and clinical characteristics between COVID-19 survivors and healthy controls were assessed using independent two sample t tests, Wilcoxon rank sum tests, and chi-square tests, as appropriate. Analyses of covariance (ANCOVA) were used to compare total and subscale SIS score differences between COVID-19 survivors and healthy controls after controlling for other variables on which there were significant group differences.

Associations of demographic and clinical characteristics with SIS total scores were examined using t tests and analysis of variance (ANOVA). A generalized linear model (GLM) was used to explore independent correlates of SIS total scores (the dependent variable) based on measures on which there were significant group differences in univariate analyses (independent variables). Significance level was set at 0.05 (two-tailed tests).

## Results

### Demographic and clinical characteristics

A total of 158 COVID-19 survivors were invited, of whom, 154 agreed to participate and completed the assessment. In addition, 194 healthy controls were recruited during the study period. Table 1 shows that there were significant differences between COVID-19 survivors and healthy controls in terms of gender, age, education level, employment status, having family members infected with COVID-19, perceptions of online mental services as helpful or not, social media use frequency, economic loss during COVID-19 pandemic, perceived economic status, perceived health status, fatigue total scores, SIS total scores and SIS subscale scores (all *p* values <0.05).

**Table 1:** Demographic and clinical characteristics of the study sample

	Healthy controls (*N* = 194)		COVID-19 survivors (*N* = 154)		Statistics		
	*n*	*%*	*N*	*%*	*x*^*2*^	*df*	*p*
Male gender	40	20.6	67	43.5	21.12	1	< 0.001
Married	133	68.6	99	64.3	0.70	1	0.40
College and above	156	80.4	69	44.8	47.63	1	< 0.001
Living with family	148	76.3	119	77.3	0.05	1	0.83
Unemployed	10	5.2	35	22.7	23.55	1	< 0.001
Family members infected with COVID-19	5	2.6	105	68.2	170.92	1	< 0.001
Feel online mental service helpful	65	33.5	20	13.0	19.58	1	< 0.001
Obvious economic loss during the COVID-19 pandemic	25	12.9	59	38.3	30.31	1	< 0.001
Frequent use of social media	132	68.0	77	50.0	11.65	1	< 0.001
Perceived economic status					22.59	2	< 0.001
Poor	38	19.6	66	42.9			
Fair	147	75.8	81	52.6			
Good	9	4.6	7	4.5			
Perceived health status					8.09	2	0.018
Poor	5	2.6	13	8.4			
Fair	88	45.3	77	50.0			
Good	101	52.1	64	41.6			
	Mean	SD	Mean	SD	*t / Z*	*df*	*p*
Age (years)	35.7	9.0	42.2	13.7	5.03	252.13 ^a^	< 0.001
Fatigue total score	3.8	2.3	2.3	2.3	6.23	— ^b^	< 0.001
PHQ-9 total score	5.3	5.3	6.2	6.0	1.35	— ^b^	0.18
Overall stigma	46.0	14.0	70.2	12.9	16.58	346	< 0.001
Social rejection	16.7	5.6	27.5	5.9	17.35	346	< 0.001
Financial insecurity	6.9	1.9	8.9	2.2	9.03	298.22 ^a^	< 0.001
Internalized shame	9.3	3.3	15.1	2.7	17.65	345.58 ^a^	< 0.001
Social isolation	13.2	4.3	18.7	4.0	12.37	346	< 0.001

*Abbreviations*: *COVID-19* coronavirus disease 2019, *SD* standard deviation, *QOL* quality of life, *PHQ-9* patient health questionnaire – 9 item, *SIS* social impact scale

a: Satterthwaite corrected because of the heterogeneity of variance

b: Wilcoxon rank sum test

After adjusting for covariates, COVID-19 survivors reported comparatively higher overall stigma levels (*F*_(1,347)_ = 60.82, *p* < 0.001), and stigma in domains of social rejection (*F*_(1,347)_ = 56.54, *p* < 0.001), financial insecurity (*F*_(1,347)_ = 19.96, *p* < 0.001), internalized shame (*F*_(1,347)_ = 71.40, *p* < 0.001) and social isolation (*F*_(1,347)_ = 34.73, *p* < 0.001).

### Correlates of overall stigma on COVID-19

**Fig. 1:**
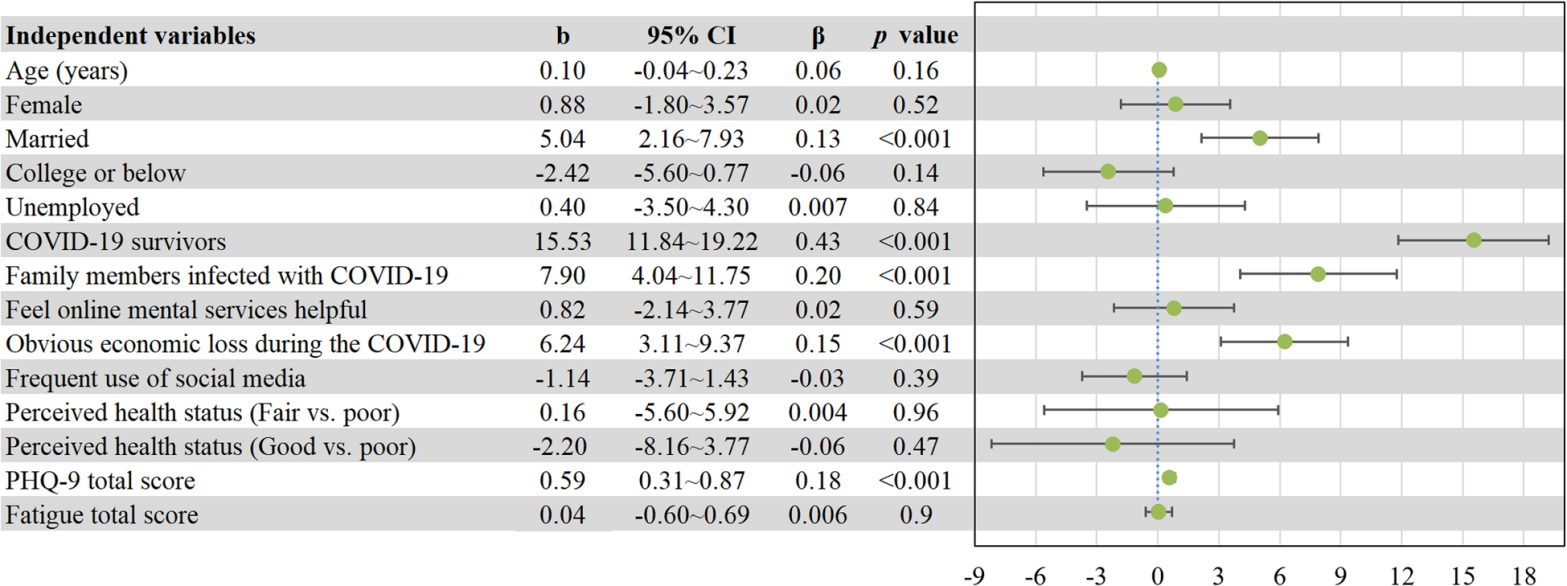
Results of generalized linear model. Abbreviations: COVID-19: coronavirus disease 2019; PHQ-9: patient health questionnaire - 9 item; b: unstandardized regression coefficient; CI: confidence interval; β: standardized regression coefficient

Univariate analyses revealed that being COVID-19 survivors, having family members infected with COVID-19, gender, marital status, education level, employment status, perceptions of online mental service, economic loss, frequency of social media use and perceived health status were significantly associated with overall stigma (all *p* values < 0.05; Table 2). GLM analysis revealed that, apart from being COVID-19 survivors, having family members infected with COVID-19, married marital status, economic loss during the COVID-19 pandemic, and depressive symptoms were positively associated with greater overall stigma (all *p* values < 0.05; Fig. 1).

**Table 2:** Overall stigma by demographic characteristics in the whole sample (*N* = 348)

Demographics	SIS total		Statistics		
	Mean	*SD*	*t / F*	*df*	*p*
Gender			2.37	346	0.018
Female	55.2	18.1			
Male	60.2	17.7			
Marital status			2.75	346	0.006
Married	58.6	17.6			
Others	53.0	18.6			
Education level			8.39	346	< 0.001
College and above	51.2	16.7			
High school and below	66.8	16.1			
Living with family			0.08	346	0.93
Yes	56.8	17.9			
No	56.6	18.8			
Occupation			4.93	346	< 0.001
Unemployed	68.7	14.7			
Employed	54.9	17.9			
Family members infected with COVID-19			17.77	277.83 ^a^	< 0.001
Yes	74.0	11.0			
No	48.7	14.8			
Feel online mental service helpful			2.42	346	0.016
Yes	52.6	17.9			
No	58.1	18.0			
Economic loss			8.20	346	< 0.001
Yes	69.7	18.0			
No	52.6	16.1			
Frequent use of social media			2.97	346	0.003
Yes	54.4	18.7			
No	60.2	16.6			
Perceived economic status			1.24	2	0.27
Poor	67.8	18.4			
Fair	52.1	15.8			
Good	50.4	16.2			
Perceived health status			14.70	2	< 0.001
Poor	71.2	17.3			
Fair	59.8	18.0			
Good	52.1	16.9			

*Abbreviations*: *COVID-19* coronavirus disease 2019, *SD* standard deviation, *SIS* social impact scale

a: Satterthwaite corrected because of the heterogeneity of variance

## Discussion

To the best of our knowledge, this is the first study to compare differences in perceived stigma between COVID-19 survivors and healthy controls. We found that COVID-19 survivors experience significantly more overall stigma and heightened stigma in domains of social rejection, financial insecurity, internalized shame and social isolation compared with healthy controls. Our findings were similar to those based on previous outbreaks of severe infectious diseases such as SARS and the Ebola virus disease (EVD) [44]. Higher levels of perceived stigma in COVID-19 survivors could be attributed to worrying about transmitting the virus to family members, friends and colleagues and fear of being discriminated against or mistreated. Fear of transmission and discrimination may deter COVID-19 survivors from disclosing their infection status or history [10].

In this study, people who had COVID-19-infected family members also reported higher levels of stigma than those without, similar to evidence from Hubei residents indicating those who had quarantined friends were more likely to experience serious scrutiny and stigma during the COVID-19 outbreak [17]. The relationship between marital status and stigma has been mixed in the infectious disease literature. Married people reported higher levels of stigma than those who were unmarried, which is consistent with earlier findings from Singapore during the SARS outbreak wherein married healthcare workers perceived more severe social stigma than did unmarried peers and tended to believe that “people avoid my family because of my job” [32]. Nevertheless, no significant associations between marital status and stigma was found during outbreaks of other infectious diseases such as EVD [27, 59].

Previous research [17] found that Hubei residents, tuberculosis patients [1] and HIV patients [6], with higher socioeconomic status were less likely to report illness-related stigma. In line with these findings, people who had greater economic losses during the COVID-19 outbreak reported higher levels of stigma. Depression was also an independent correlate of COVID-19-related stigma in this study, consistent with evidence from the SARS outbreak [62] and HIV-positive patients [24, 38]. This relationship may be a partial reflection of shared core symptoms between depression and stigma including feelings of guilt, self-blame and low self-esteem [12, 52]. In addition, the relationship between depression and stigma is considered to be bi-directional based on several theoretical models of stigma and infectious disease or disability; i.e., stigma could increase the risk of depression, and vice versa [24, 36, 55, 60, 73].

The association between education level and infectious disease related stigma has been inconsistent between studies. For instance, some studies found that lower education is associated with increased stigma in patients with HIV [6], tuberculosis [1] or EVD [30] while other studies found complementary associations in patients with COVID-19 [17] or EVD [59]. James et al. found that educational level was not significantly associated with internalized or enacted stigma among Ebola survivors, paralleling results of this study [27].

Previous studies have also found that exposure to social media could help reduce HIV-related stigma [3, 7, 45]. However, we did not find significant associations between social media use / online mental services and COVID-19-related stigma. Our findings were similar to those of Duan et al. [17] who also reported an absence of such relations. Presumably, if social media releases misinformation and stigmatizing viewpoints to the lay public, the positive influence of media exposure on stigma could be offset [34, 64]. This assumption appears to have merit in light of evidence that misinformation and stigmatizing viewpoints have been common in social media during the COVID-19 pandemic, particularly in its early stages [21, 37].,

There are several methodological limitations in this study that should be acknowledged. First, because this was a cross-sectional study, causal relations between stigma and other variables could not be examined. Second, for logistical reasons, close contacts and family members of persons with COVID-19 cases were not analyzed separately. Third, although results documented correlates of perceived stigma, external stigma was not assessed in this study [1, 46, 65, 71]. Finally, convenience sampling of unmatched healthy controls from the community was undertaken to facilitate rapid data collection within the relatively narrow time window of the pandemic in which COVID-19 patients were also recruited. Consequently, the control group was unlikely to be representative of the population from which they were drawn and generalizations cannot be made from our sample to the uninfected population of Chongqing residents. Fortunately, key demographic measures were assessed in this study and potential confounding effects of group differences in demographics were statistically controlled within main analyses.

To reduce COVID-19-related stigma, health authorities and academic associations in many countries and territories have released appeals to stop stigmatizing at-risk groups including COVID-19 survivors, people returning from overseas and those released from quarantine [11, 41, 70]. These public health messages highlight negative consequences of stigma (e.g., reluctance to disclose infected status or seek assessment and treatment) that undermine efforts to treat the disease and mitigate further community spread [41]. Stigma also increases risks of fear, anger, discrimination, and violence toward ordinary people instead of devoting resources to eradicating the disease itself [11]. Underscoring the importance of communications to reduce stigma, the Mayo Clinic released a call entitled “What you can do to reduce COVID-19 stigma”, appealing for the public to access COVID-19 information from reputable sources such as the Centers for Disease Control and Prevention (CDC) and WHO, and provide support for people who may feel stigmatized [41]. CDC appeals have been made to community leaders and public health officials to prevent stigma by maintaining the privacy and confidentiality of COVID-19 survivors, correcting negative language that can cause stigma, and using media to challenge stereotyping and stigmatization [11]. The Washington State Department of Health has set up channels to report discrimination among those who encounter stigmatization or discrimination [63]. In China, some public media has also issued commentaries appealing to stop stigmatizing COVID-19 survivors [49]. In light of our results, continued public health appeals devoted to reducing COVID-19 stigma in China are clearly warranted.

## Conclusion

In conclusion, COVID-19-related stigma is commonly experienced among COVID-19 survivors even though the outbreak has been well-contained in China. In light of our findings linking the COVID-19 diagnosis, economic losses, and depression with elevations in perceived stigma, routine assessment of perceived stigma should be conducted on COVID-19 survivors and other at-risk groups. Appropriate psychological assistance should be provided to those distressed by these experiences. In addition, science-based public health education and anti-stigma / anti-discrimination policies should be enacted and enforced in legal legislation to reduce stigmatizing responses of social institutions and the general public. Finally, the long-term impact of the COVID-19 pandemic on stigma should be examined in future studies with prospective research designs.

## Data Availability

The Clinical Research Ethics Committee of Chongqing Mental Health Center that approved the study prohibits the authors from making the research data set publicly available. Readers and all interested researchers may contact Dr. Guo-Qing Jiang (Email address: 1159424975@qq.com) for details. Dr. Jiang could apply to the Clinical Research Ethics Committee of Chongqing Mental Health Center for the release of the data.

## Abbreviations

SIS: Social Impact Scale
ANCOVA: Analysis of covariance
ANOVA: Analysis of variance
b: Unstandardized regression coefficient
β: Standardized regression coefficient
COVID-19: Coronavirus disease 2019
CI: Confidence interval
EVD: Ebola virus disease
GLM: Generalized linear model
HIV: Human immunodeficiency virus
PHQ-9: Patient health questionnaire - 9 item
SARS: Severe acute respiratory syndrome
SAS: Statistical Analysis System

## References

[1] Abioye I, Omotayo M, Alakija W (2011). Socio-demographic determinants of stigma among patients with pulmonary tuberculosis in Lagos, Nigeria. Afr Health Sci.

[2] Adom, D., Adu Mensah, J., 2020. The psychological distress and mental health disorders from COVID-19 stigmatization in Ghana. Social sciences & humanities open, available at SSRN: https://ssrn.com/abstract=3599756 or 10.2139/ssrn.3599756 (access 13 May 2020). 10.1016/j.ssaho.2021.100186 PMC8257423 34250461

[3] Aghaei A, Mohraz M, Shamshirband S (2020). Effects of media, interpersonal communication and religious attitudes on HIV-related stigma in Tehran, Iran. Informatics in Medicine Unlocked.

[4] American Psychological Association, 2019. Discrimination: What it is, and how to cope. https://www.apa.org/topics/discrimination (access 31 Oct 2019).

[5] Amnesty International, 2019. What drives discrimination and how do we stop it? , https://www.amnesty.org/en/what-we-do/discrimination/ (access 13 Oct 2020).

[6] Amuri M, Mitchell S, Cockcroft A, Andersson N (2011). Socio-economic status and HIV/AIDS stigma in Tanzania. AIDS Care.

[7] Babalola S, Fatusi A, Anyanti J (2009). Media saturation, communication exposure and HIV stigma in Nigeria. Soc Sci Med.

[8] Bagcchi S (2020). Stigma during the COVID-19 pandemic. Lancet Infect Dis.

[9] Baldassarre A, Giorgi G, Alessio F, Lulli LG, Arcangeli G, Mucci N (2020). Stigma and discrimination (SAD) at the time of the SARS-CoV-2 pandemic. Int J Environ Res Public Health.

[10] Bu, N.-N., 2020. The 80 thousand COVID-19 survivers are undergoing discriminaton (in Chinese). http://k.sina.com.cn/article_1690367810_64c0f74201900py8d.html?from=mood (access 18 July 2020).

[11] Centers for Disease Control and Prevention, 2020. Reducing Stigma. https://www.cdc.gov/coronavirus/2019-ncov/daily-life-coping/reducing-stigma.html (access 11 June 2020).

[12] Charles B, Jeyaseelan L, Pandian AK, Sam AE, Thenmozhi M, Jayaseelan V (2012). Association between stigma, depression and quality of life of people living with HIV/AIDS (PLHA) in South India - a community based cross sectional study. BMC Public Health.

[13] China Daily, 2021. Latest data on novel coronavirus. https://www.chinadaily.com.cn/china/special_coverage/2020latestdata (access 21 Mar 2021).

[14] Cooke GS, Andrieux-Meyer I, Applegate TL, Atun R, Burry JR, Cheinquer H, Dusheiko G, Feld JJ, Gore C, Griswold MG, Hamid S, Hellard ME, Hou J, Howell J, Jia J, Kravchenko N, Lazarus JV, Lemoine M, Lesi OA, Maistat L, McMahon BJ, Razavi H, Roberts T, Simmons B, Sonderup MW, Spearman CW, Taylor BE, Thomas DL, Waked I, Ward JW, Wiktor SZ (2019). Accelerating the elimination of viral hepatitis: a Lancet Gastroenterology & Hepatology Commission. Lancet Gastroenterol Hepatol.

[15] DingXiangYiSheng, 2021. COVID-19 Global Pandemic Real-time Report. https://ncov.dxy.cn/ncovh5/view/en_pneumonia?from=dxy&source=&link=&share= (access 26 Feb 2021).

[16] Do Duy C, Nong VM, Van AN, Thu TD, Do Thu N, Quang TN (2020). COVID-19 related stigma and its association with mental health of health-care workers after quarantined in Vietnam. Psychiatry Clin Neurosci.

[17] Duan W, Bu H, Chen Z (2020). COVID-19-related stigma profiles and risk factors among people who are at high risk of contagion. Soc Sci Med.

[18] Dubey S, Biswas P, Ghosh R, Chatterjee S, Dubey MJ, Chatterjee S, Lahiri D, Lavie CJ (2020). Psychosocial impact of COVID-19. Diab Metabol Syndrome.

[19] Earnshaw VA, Brousseau NM, Hill EC, Kalichman SC, Eaton LA, Fox AB (2020). Anticipated stigma, stereotypes, and COVID-19 testing. Stigma Health.

[20] Fife BL, Wright ER (2000). The dimensionality of stigma: a comparison of its impact on the self of persons with HIV/AIDS and cancer. J Health Soc Behav.

[21] Geldsetzer P (2020). Knowledge and perceptions of COVID-19 among the general public in the United States and the United Kingdom: a cross-sectional online survey. Ann Intern Med.

[22] Gladman D, Nash P, Goto H, Birt JA, Lin CY, Orbai AM, Kvien TK (2020). Fatigue numeric rating scale validity, discrimination and responder definition in patients with psoriatic arthritis. RMD Open.

[23] Goffman, E., 1963. Stigma: notes on the management of spoiled identity. Prentice-Hall, United Kingdom.

[24] Grov C, Golub SA, Parsons JT, Brennan M, Karpiak SE (2010). Loneliness and HIV-related stigma explain depression among older HIV-positive adults. AIDS Care.

[25] Grover S, Singh P, Sahoo S, Mehra A (2020). Stigma related to COVID-19 infection: are the health care workers stigmatizing their own colleagues?. Asian J Psychiatr.

[26] Gu Y, Zhu Y, Xu F, Xi J, Xu G. Factors associated with mental health outcomes among patients with COVID-19 treated in the Fangcang shelter hospital in China. Asia Pac Psychiatr. 2020:e12443. 10.1111/appy.12443. 10.1111/appy.12443 33135397

[27] James PB, Wardle J, Steel A, Adams J (2020). An assessment of Ebola-related stigma and its association with informal healthcare utilisation among Ebola survivors in Sierra Leone: a cross-sectional study. BMC Public Health.

[28] Johns Hopkins University (2020). COVID-19 Dashboard by the Center for Systems Science and Engineering (CSSE) at Johns Hopkins University (JHU).

[29] Kaufman KR, Petkova E, Bhui KS, Schulze TG (2020). A global needs assessment in times of a global crisis: world psychiatry response to the COVID-19 pandemic. BJPsych Open.

[30] Kelly JD, Weiser SD, Wilson B, Cooper JB, Glayweon M, Sneller MC, Drew C, Steward WT, Reilly C, Johnson K, Fallah MP (2019). Ebola virus disease-related stigma among survivors declined in Liberia over an 18-month, post-outbreak period: an observational cohort study. PLoS Negl Trop Dis.

[31] Kılınçel Ş, Kılınçel O, Muratdağı G, Aydın A, Usta MB. Factors affecting the anxiety levels of adolescents in home-quarantine during COVID-19 pandemic in Turkey. Asia Pac Psychiatr. 2020:e12406. 10.1111/appy.12406. 10.1111/appy.12406 PMC7435562 32783389

[32] Koh D, Lim MK, Chia SE, Ko SM, Qian F, Ng V, Tan BH, Wong KS, Chew WM, Tang HK, Ng W, Muttakin Z, Emmanuel S, Fong NP, Koh G, Kwa CT, Tan KB, Fones C (2005). Risk perception and impact of severe acute respiratory syndrome (SARS) on work and personal lives of healthcare workers in Singapore: what can we learn?. Med Care.

[33] Koschorke M, Evans-Lacko S, Sartorius N, Thornicroft G, Gaebel W, Rössler W, Sartorius N (2017). Stigma in Different Cultures. The stigma of mental illness - end of the story?.

[34] Lee S, Chan LY, Chau AM, Kwok KP, Kleinman A (2005). The experience of SARS-related stigma at Amoy gardens. Soc Sci Med.

[35] Levin S, Van Laar C (2006). Stigma and group inequality: social psychological perspectives.

[36] Li L, Lee SJ, Thammawijaya P, Jiraphongsa C, Rotheram-Borus MJ (2009). Stigma, social support, and depression among people living with HIV in Thailand. AIDS Care.

[37] Lin C-Y (2020). Social reaction toward the 2019 novel coronavirus (COVID-19). Social Health and Behavior.

[38] Logie C, James L, Tharao W, Loutfy M (2013). Associations between HIV-related stigma, racial discrimination, gender discrimination, and depression among HIV-positive African, Caribbean, and black women in Ontario, Canada. AIDS Patient Care STDs.

[39] Ma Y, Zhan N. To mask or not to mask amid the COVID-19 pandemic: how Chinese students in America experience and cope with stigma. Chinese Sociological Review. 2020:1–26. 10.1080/21620555.2020.1833712.

[40] Mak WW, Cheung F, Woo J, Lee D, Li P, Chan KS, Tam CM (2009). A comparative study of the stigma associated with infectious diseases (SARS, AIDS, TB). Hong Kong Med J.

[41] Mayo Clinic, 2020. COVID-19 (coronavirus) stigma: what it is and how to reduce it., https://www.mayoclinic.org/diseases-conditions/coronavirus/in-depth/coronavirus-stigma/art-20484278 (access 17 Apr 2020).

[42] Menon V, Padhy SK, Pattnaik JI (2020). Stigma and aggression against health Care Workers in India Amidst COVID-19 times: possible drivers and mitigation strategies. Indian J Psychol Med.

[43] Misra S, Le PD, Goldmann E, Yang LH (2020). Psychological impact of anti-Asian stigma due to the COVID-19 pandemic: a call for research, practice, and policy responses. Psychol Trauma Theory Res Pract Policy.

[44] Muhidin S, Vizheh M, Moghadam ZB (2020). Anticipating COVID-19-related stigma in survivors and health-care workers: lessons from previous infectious diseases outbreaks - an integrative literature review. Psychiatry Clin Neurosci.

[45] O'Leary A, Kennedy M, Pappas-DeLuca KA, Nkete M, Beck V, Galavotti C (2007). Association between exposure to an HIV story line in the bold and the beautiful and HIV-related stigma in Botswana. AIDS Educ Prev.

[46] Overholt L, Wohl DA, Fischer WA, Westreich D, Tozay S, Reeves E, Pewu K, Adjasso D, Hoover D, Merenbloom C, Johnson H, Williams G, Conneh T, Diggs J, Buller A, McMillian D, Hawks D, Dube K, Brown J (2018). Stigma and Ebola survivorship in Liberia: results from a longitudinal cohort study. PLoS One.

[47] Pan AW, Chung L, Fife BL, Hsiung PC (2007). Evaluation of the psychometrics of the social impact scale: a measure of stigmatization. Int J Rehabil Res.

[48] Pan X, Xiao Y, Ren D, Xu ZM, Zhang Q, Yang LY, et al. Prevalence of mental health problems and associated risk factors among military healthcare workers in specialized COVID-19 hospitals in Wuhan, China: A cross-sectional survey. Asia Pac Psychiatr. 2020:e12427. 10.1111/appy.12427. 10.1111/appy.12427 PMC7645907 33089622

[49] People's Daily, 2020. It is unhealthy to stigmatize COVID-19 survivors (in Chinese). https://baijiahao.baidu.com/s?id=1660318823130857224&wfr=spider&for=pc (access 5 Mar 2020).

[50] Peprah P, Gyasi RM (2020). Stigma and COVID-19 crisis: a wake-up call. The International Journal of Health Planning and Management.

[51] Ramaci T, Barattucci M, Ledda C, Rapisarda V (2020). Social stigma during COVID-19 and its impact on HCWs outcomes. Sustainability.

[52] Roeloffs C, Sherbourne C, Unützer J, Fink A, Tang L, Wells KB (2003). Stigma and depression among primary care patients. Gen Hosp Psychiatry.

[53] Salari N, Hosseinian-Far A, Jalali R, Vaisi-Raygani A, Rasoulpoor S, Mohammadi M, Rasoulpoor S, Khaledi-Paveh B (2020). Prevalence of stress, anxiety, depression among the general population during the COVID-19 pandemic: a systematic review and meta-analysis. Glob Health.

[54] Salazar de Pablo G, Vaquerizo-Serrano J, Catalan A, Arango C, Moreno C, Ferre F, Shin JI, Sullivan S, Brondino N, Solmi M, Fusar-Poli P (2020). Impact of coronavirus syndromes on physical and mental health of health care workers: systematic review and meta-analysis. J Affect Disord.

[55] Shin J-S, Lee K-H, Kim K-S, Lee Y-I (2011). The impact of perceived social stigma on depression among people with disabilities living in Chungbuk. J Community Welfare.

[56] Shoib S, Nagendrappa S, Grigo O, Rehman S, Ransing R (2020). Factors associated with COVID-19 outbreak-related suicides in India. Asian J Psychiatr.

[57] Siu JY (2008). The SARS-associated stigma of SARS victims in the post-SARS era of Hong Kong. Qual Health Res.

[58] Spitzer RL, Kroenke K, Williams JB (1999). Validation and utility of a self-report version of PRIME-MD: the PHQ primary care study. Primary care evaluation of mental disorders. Patient Health Questionnaire. Jama.

[59] Tenkorang EY (2017). Ebola-related stigma in Ghana: individual and community level determinants. Soc Sci Med.

[60] Turan B, Smith W, Cohen MH, Wilson TE, Adimora AA, Merenstein D, Adedimeji A, Wentz EL, Foster AG, Metsch L, Tien PC, Weiser SD, Turan JM (2016). Mechanisms for the negative effects of internalized HIV-related stigma on antiretroviral therapy adherence in women: the mediating roles of social isolation and depression. J Acquir Immune Defic Syndr.

[61] Venugopal, V.C., Mohan, A., Chennabasappa, L.K., 2020. Status of mental health and its associated factors among the general populace of India during COVID-19 pandemic. Asia Pac psychiatry, e12412. 10.1111/appy.12412. 10.1111/appy.12412 PMC7460994 32830876

[62] Verma S, Mythily S, Chan YH, Deslypere JP, Teo EK, Chong SA (2004). Post-SARS psychological morbidity and stigma among general practitioners and traditional Chinese medicine practitioners in Singapore. Ann Acad Med Singap.

[63] Washington State Department of Health, 2020. Stigma Reduction. https://www.doh.wa.gov/CommunityandEnvironment/HealthEquity/Stigma/COVID19StigmaReduction (access 28 Feb 2021).

[64] Williams J, Gonzalez-Medina D (2011). Infectious diseases and social stigma. Applied Innovations and Technologies.

[65] Wolitski RJ, Pals SL, Kidder DP, Courtenay-Quirk C, Holtgrave DR (2009). The effects of HIV stigma on health, disclosure of HIV status, and risk behavior of homeless and unstably housed persons living with HIV. AIDS Behav.

[66] World Health Organization (2020). Naming the coronavirus disease (COVID-19) and the virus that causes it.

[67] World Health Organization (2020). Novel Coronavirus – China.

[68] World Health Organization, 2020c. When talking about #COVID19, certain words & language may have a negative meaning for people and fuel stigmatizing attitudes [Twitter post]. https://twitter.com/WHO/status/1234597035275362309 (access 2 Mar 2020).

[69] World Health Organization, 2020d. WHO Director-General's opening remarks at the media briefing on COVID-19 - 11 March 2020. https://www.who.int/dg/speeches/detail/who-director-general-s-opening-remarks-at-the-media-briefing-on-covid-19%2D%2D-11-march-2020 (access 11 Mar 2020).

[70] World Health Organization, 2020e. A guide to preventing and addressing social stigma associated with COVID-19. https://www.who.int/publications/m/item/a-guide-to-preventing-and-addressing-social-stigma-associated-with-covid-19?gclid=EAIaIQobChMIjaPxkeCL17wIVzKiWCh13mMgS17EAAYASAAEgL13o_D_BwE (access 24 Feb 2020).

[71] Wright K, Naar-King S, Lam P, Templin T, Frey M (2007). Stigma scale revised: reliability and validity of a brief measure of stigma for HIV+ youth. J Adolesc Health.

[72] Xu Y, Wu HS, Xu YF (2007). The application of patient health questionnaire 9 in community elderly population: reliability and validity. Shanghai Archives Psychiatr.

[73] Yıldırım Z, Ertem DH, Ceyhan Dirican A, Baybaş S (2018). Stigma accounts for depression in patients with epilepsy. Epilepsy Behav.

[74] Zandifar A, Badrfam R, Khonsari NM, Mohammadi MR, Asayesh H, Qorbani M (2020). Prevalence and associated factors of posttraumatic stress symptoms and stigma among health care workers in contact with COVID-19 patients. Iran J Psychiatry.

